# Clinical Implication of Toll-Like Receptors (*TLR2* and* TLR4*) in Acute Myeloid Leukemia Patients

**DOI:** 10.31557/APJCP.2020.21.11.3177

**Published:** 2020-11

**Authors:** Salah Aref, AL Shaimaa Abd Elmaksoud, Sherin Abd Elaziz, Mohamed Mabed, Mohamed Ayed

**Affiliations:** 1 *Hematology Unit, Department of Clinical pathology, Mansoura Faculty of Medicine, Mansoura University, Egypt. *; 2 *Hematology Unit, Mansoura University Oncology Center, Mansoura University, Egypt. *

**Keywords:** TLR2, TLR4, polymorphisms, AML

## Abstract

**Backgrounds:**

Toll-like receptors 2; 4 (TLR2;4) are an essential component of the innate immunity and play an important role in immune-surveillance and immune response to various microorganisms. This study aimed to investigate the association between *TLR2* and* TLR4* polymorphism and the risk of acquiring severe infections, and impact on AML patient’s outcome.

**Subjects and methods:**

Using polymerase chain reaction - restriction fragment length polymorphism (PCR-RFLP); we analyzed three SNPs in the *TLR2* (*Arg753Gln*) and *TLR4* (*Asp299Gly* and *Thr399Ile*) in 120 AML patients and 100 healthy control subjects.

**Results:**

No significant differences in genotype or alleles frequency between healthy controls and AML patients regarding *TLR2 Arg753Gln*, *TLR4 Asp299Gly* and *TLR4 Thr399Ile* polymorphisms (P>0.05 for all). Neutropenic fever was detected in 110 out of 120 (91.7%) of the studied AML patients. The sepsis and pneumonia were identified in 20 out of 120 patients (16.7%). The incidence of sepsis was associated with *TLR2 Arg753Gln*:* AG* genotypes, A allele and *TLR4 Asp299Gly*: CT genotype and C allele as compared to other genotypes and alleles. Moreover; *TLR2 (Arg753Gln) GG *polymorphisms significantly associated with shortest overall survival (OS) and shortest disease-free survival (DFS); while *TLR4* polymorphisms affect the DSF only but not OS. In AML patients *TLR2 Arg753Gln* gene polymorphism is associated with high susceptibility to sepsis and TLR4 (*Asp299Gly and Thr399Ile*) gene polymorphism is associated with high susceptibility for both pneumonia; and sepsis.

**Conclusion:**

*TLR2 Arg753Gln *(*AG; GG* genotype) polymorphisms are associated with shortest OS and DFS. Moreover; significant association between *TLR2* polymorphisms, *TLR4 Arg753Gln* polymorphisms and risk of severe infections in AML patients was documented.

## Introduction

Toll-like receptors (*TLRs*) are a family of transmembrane receptors which play an important role in the host defense against microorganisms. *TLRs* are mainly expressed in human immune-related cells such as monocytes, neutrophils, macrophages, dendritic cells, T cells, B cells and NK cells (Uematsu et al., 2006; Rybka et al., 2015). TLRs alert the immune system to infection by recognizing pathogen-associated molecular patterns derived from various microorganisms (Beutler et al., 2004a; Beutler et al., 2004b). Furthermore, functional TLRs are expressed not only in immune cells, but also in cancer cells, thus implicating a role of TLRs in tumor biology (Zhu et al., 2013). About eleven human TLRs have been identified up till now and each of them participates in a specific intracellular signaling pathway. TLRs 1, 2, 4, 5 and 6 are typical for bacterial products, TLRs 3, 7 and 8 are characteristic for viral infection and TLR9 is associated with bacterial and viral inflammatory response (Tsujimoto et al., 2008). *TLR2* and *TLR4* are two of the most studied TLRs to have an important role in the recognition of both bacterial and fungal pathogens (Netea, et al., 2007).

Leukemia is a type of blood cancer that results from an abnormal functioning of the bone marrow, which tends to cause an abnormal proliferation of white or red blood cells. It may occur in acute or chronic form (Yamaguti et al., 2010; Aref et al., 2020). Induction chemotherapy of acute leukemia results in a long period of neutropenia that is associated with a high risk of infectious complications (Syrjälä et al., 2010). Infectious complications continue to be one of the major causes of morbidity and mortality in patients with acute leukemia (Schnetzke et al., 2015). Sepsis and pneumonia represent the most important infectious complications and are associated with an increased mortality after induction chemotherapy (Hamalainen et al., 2008). The occurrence of infectious complications represents a crucial factor affecting the prognosis of acute leukemia patients, so prophylaxis and treatment of infections have an important role in the clinical management of these patients (Gupta et al., 2010).

Sepsis is a complex clinical syndrome that results from a systemic inflammatory response to bacteria and/or bacterial products (Uematsu et al., 2006). It remains a leading cause of death in the non-cardiac intensive care unit (ICU) despite improvements in antibiotic therapy and supportive care

Only a few retrospective analyses investigate whether polymorphisms of innate immunity influence the risk of severe infections in acute leukemia patients. Importantly, these studies do not demonstrate any correlation between different SNPs and important clinical events such as fungal infections or sepsis (Schnetzke et al., 2015).

The Expression of Toll-like Receptors in Patients with Acute Myeloid Leukemia Treated With Induction Chemotherapy have been studied (Rybka et al., 2015; Ramzi et al., 2018); however the impact of Toll like receptors 2, 4 polymorphism on the AML patients outcome are not previously studied.

In this study, we investigated the impact of *TLR2* and TLR4 SNPs on susceptibility of AML patients towards developing sepsis, pneumonia and fever during severe neutropenia following intensive chemotherapy as well as its impact on the patient’s outcome.

*Subjects*

This is a case control study including 120 patients with newly diagnosed acute myeloid leukemia (AML) (64 male, 56 females; median age 48 years, range 18–77 years) and 100 normal healthy controls (46 males, 54 females; median age 47 years, with age range between 26–62 years.). The study was approved by the Institutional Revision Board (IRB) of Mansoura University. Patients with secondary AML, and pediatric AML were excluded from this study. The AML diagnosis was performed according to the WHO criteria. The AML FAB subtypes include M0; M1; M2, M3, M5 and M5. 

All patients were treated with induction chemotherapy including anthracyclines and arabinoside cytosine. Prophylactic oral antibiotics (covering both gram positive and negative organisms) were used in all patients. A total of 30 patients (25%) had favorable cytogenetic/ molecular risk, 55 (45.8%) patients had intermediate cytogenetic/molecular risk and 35 patients (29.2 %) had poor cytogenetic/molecular risk. Bone marrow samples were taken before induction therapy. Samples were collected after informed consent from patients and control group.

All patients enrolled in this study underwent curative-intent induction chemotherapy at Oncology Center Mansoura University, Egypt, between 2016 and 2017. The follow up period was one year for all cases. The study was approved by the Institutional Revision Board (IRB) of Mansoura University. Patients with secondary AML, and pediatric AML were excluded from this study. Clinical characteristics of all investigated AML patients are demonstrated in Table 1.

*Exclusion criteria*

Patients with secondary AML, and pediatric AML were excluded from this study.

*Follow up*

The included patients were observed for up to 12 months.

## Materials and Methods

*Analysis of TLR polymorphisms*

Genotyping of *TLR2 Arg753Gln (R753Q, rs5743708), TLR4 Asp299Gly (D299G, rs4986790)* and* TLR4 Thr399Ile (T399I, rs4986791) *was performed by polymerase chain reaction-restriction fragment length polymorphism (PCR-RFLP) method. Briefly, genomic DNA was isolated from peripheral blood or bone marrow samples using Gene *JETTM* Whole Blood Genomic DNA Purification Mini Kits from Thermo Scientific (lot no k0781, Lithuania, EU). PCR was done by amplification of extracted genomic DNA via thermal cycler (ARKTIK Thermal Cycler, Thermo Scientific Co.). *TLR4 Asp299Gly* forward primer, 5′- AGCATACTTAGACTACTACCTCCATG-3′and reverse primer: 5′- GAGAGATTTGAGTTTCAATGTGGG-3′; TLR4 Thr399Ile forward primer, 5′-GGTTGCTGTTCTCAAAGTGATTTTGGGAGAA-3′and reverse primer: 5′-GGAAATCCAGATGTTCTAGTTGTTCTAAGCC-3′; TLR2 Arg753Gln forward primer, 5′-TTGACTCCATTGAAAAGAGC-3′and reverse primer, 5′-TAAATATGGGAACCTAGGAC-3 ′. Reactions were performed in a 25 μl volume containing 12.5 μL of red master mix, 0.1μL of both forward and reverse primer, 1 μL of DNA, 11.3 μL of distilled water (DW).

One hundred nanograms of genomic DNA was amplified using the following cycling conditions for PCR of *TLR4 Asp299Gly*: 95°C for 15 min, 35 cycles of 95 °C for 30 s, 55°C for 60 s and 72 °C for 30 s followed by a final extension of 72°C for 10 min. The PCR protocol for

*TLR4 Thr399Ile* was the same as for *TLR4 Asp299Gly *except that annealing temperature was 53°C. The PCR protocol for *TLR2 Arg753Gln* was the same as for* TLR4 Asp299Gly* except that annealing temperature was 54°C for 30 s and the final extension of 72°C for 5 min. 

The PCR products were then digested at 37°C with HinfI for *Thr399Ile* polymorphism for 5-15 min (New England BioLabs Inc, lot no 0411604), NcoI for *Asp299Gly* polymorphism for 25 min (New England BioLabs Inc, lot no 0061605), PstI for *Arg753Gln *polymorphism for 5-15 min (New England BioLabs Inc, lot no 0021504). The digests were run on a 2% agarose gel and visualized under UV light using ethidium bromide. 

Digestion of PCR product for *TLR4 Asp299Gly* using NcoI enzyme produced a fragment of 190 bp for homozygous wild type, produced two fragments of 168 and 20 bp for homozygous mutant type, and produced three fragments of 190, 168 and 20 bp for heterozygous mutant type. 

Digestion of PCR product for *TLR4 Thr399Ile* using HinfI enzyme produced a fragment of 124 bp for homozygous wild type, produced two fragments of 98 and 26 bp for homozygous mutant type, and produced three fragments of 124, 98 and 26 bp for heterozygous mutant type. 

Digestion of PCR product for *TLR2 Arg753Gln *using PstI enzyme produced a fragment of 300 bp for homozygous wild type, produced two fragments of 190 and 110 bp for homozygous mutant type, and produced three fragments of 300, 190 and 110 bp for heterozygous mutant type. 

*Statistical analysis*

The statistical analysis of the data was performed by using excel (Microsoft office 2013) program and SPSS (Statistical Package for Social Science) program (SPSS, Inc, Chicago, IL) version 20. Chi square test was used to compare groups. Quantitative data were presented as median and range, mean and standard deviation. For comparison between two groups Mann-Whitney test (for non-parametric data) was used. For comparison of more than two groups; Kruskal-Wallis (for non-parametric data) was used. Univariate analysis used to evaluate association of polymorphism with occurrence of sepsis, pneumonia and fever. For survival analysis, Kaplan-Meier curves were used and compared by log-rank test. The P value<0.05 was considered statistically significant. 

**Figure 1:**
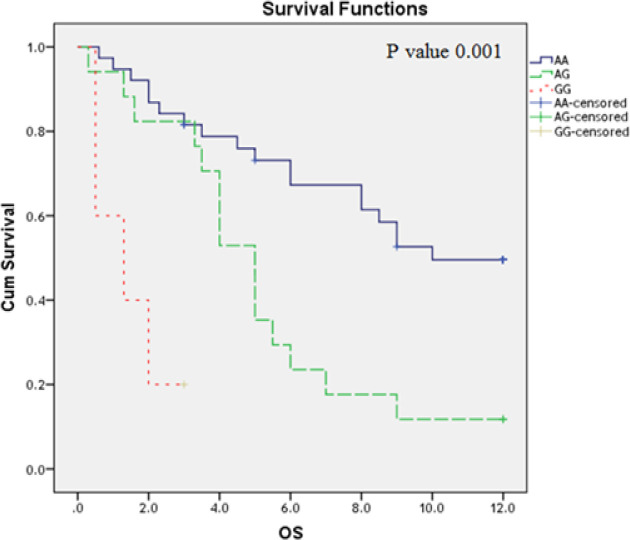
Impact of TLR2 Arg753Gln Genotype Variants on AML Overall Survival (OS) (Months)

**Figure 2:**
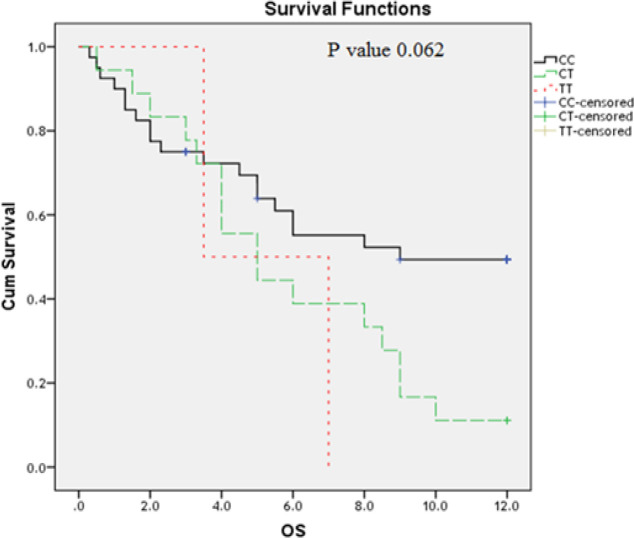
Impact of TLR4 Thr399Ile Genotype Variants on AML Overall Survival (OS) (Months)

**Table 1:** AML Patients Characteristics

Parameters	AML Patients
Age (years) median (range))	48 (18-77)
Gender	Male (n=64)
	Female (n=56)
Peripheral blood blast (%), median (range)	24.5 (0 - 90)
Bone marrow blast (%), median (range)	80.0 (23-95)
AML subtypes	AML with minimally differentiated (n=6)
	AML without maturation (n=24)
	AML with maturation (n=32)
	AML myelomonocytic (n=40)
	AML monoblastic (n=18)
Cytogenetic findings	Good ( n=30)
	Intermediate(n=55)
	Poor (n=35)
Infections status	Neutropenic fever (n=80)
	patients Sepsis (n=20)
	Pneumonia (n=20)
Causes of Infections (n= 80)	Bacterial (n=60); Fungal (n=15); Viral (n=5)
Sources of infection	Respiratory System (n=50); Abdomen (n= 20)
	Urinary tract (n= 8); Skin (n= 2)
Induction remission response	Responder (n= 74)
	Resistant (n=30)
	Partial response (n=16)
Disease Free Survival (DFS)	Relapse (n=55)
	No relapse (n=65)
AML patients Outcome	Alive (n=46)
	Dead (n= 74)

**Figure 3:**
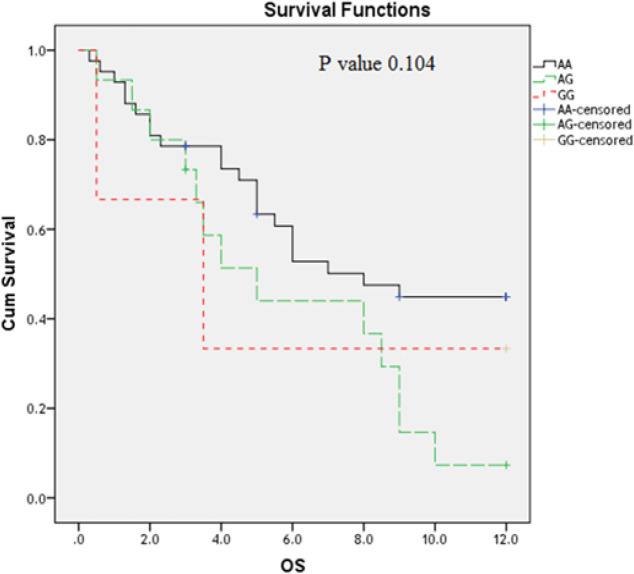
Impact of T*LR4 Asp299Gly* Genotype Variants on AML Overall Survival (OS) (Months)

**Table 2.** Distribution of *TLR2 Arg753Gln* Genotype Variants and Alleles in AML Groups Versus Control

Genotypes	Control	AML	Relative risk of AML			
	(n=100)	(n=120)	OR	95%CI		P
AA	78	78	1	-	-	R
AG	18	33	1.833	0.667	5.039	0.24
GG	4	9	2.25	0.351	14.4	0.392
A	174	189	1.805	0.818	3.985	0.143
G	26	51				

OR, odds ratio; CI, Confidence interval; Significant (P value < 0.05)

**Table 3:** Distribution of *TLR4 Thr399Ile* Genotype Variants and Alleles in ALL and AML Groups Versus Control

Genotypes	Control	AML	Relative risk of AML			
	(n=100)	(n=120)	OR	95% CI		P value
CC	82	78	1	-	-	R
CT	16	36	2.365	0.852	6.564	0.098
TT	2	6	3.153	0.272	36.55	0.358
C	180	192	2.25	0.959	5.27	0.062
T	20	48				

CI, Confidence interval; OR, odds ratio

**Figure 4:**
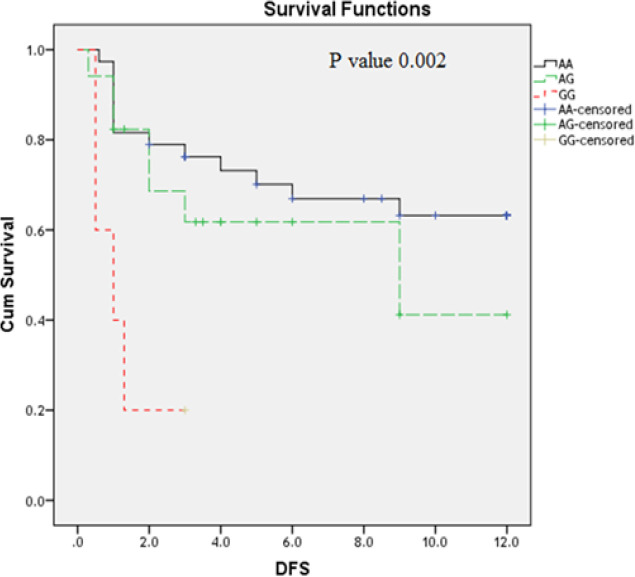
Impact of *TLR2 Arg753Gln* Genotype Variants on AML Disease Free Survival (DFS) (Months)

**Table 4:** Distribution of *TLR4 Asp299Gly* Genotype Variants and Alleles in AML Groups Versus Control

Genotypes	Control	AML	Relative risk of AML			
	(n=100)	(n=120)	OR	95% CI		P value
AA	86	84	1	-	-	R
AG	12	30	2.559	0.836	7.832	0.099
GG	2	6	3.071	0.265	35.49	0.899
A	184	198	2.439	0.967	6.148	0.058
G	16	42				

CI, Confidence interval; OR, odds ratio

**Figure 5:**
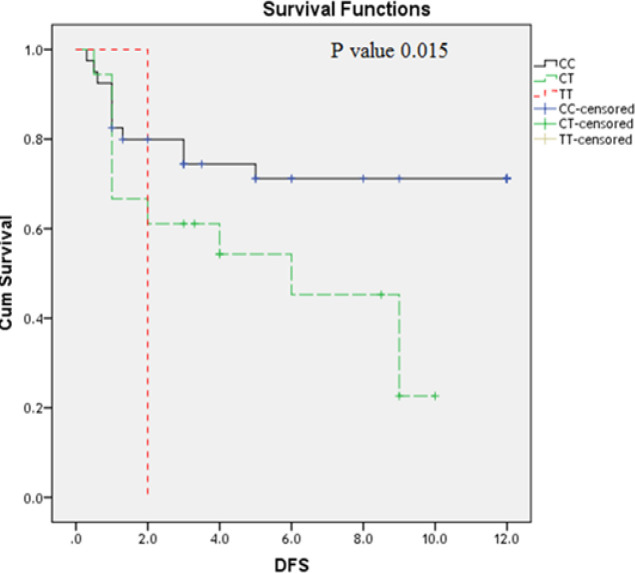
Impact of *TLR4 Thr399Ile* Genotype Variants on AML DFS (Months)

**Figure 6:**
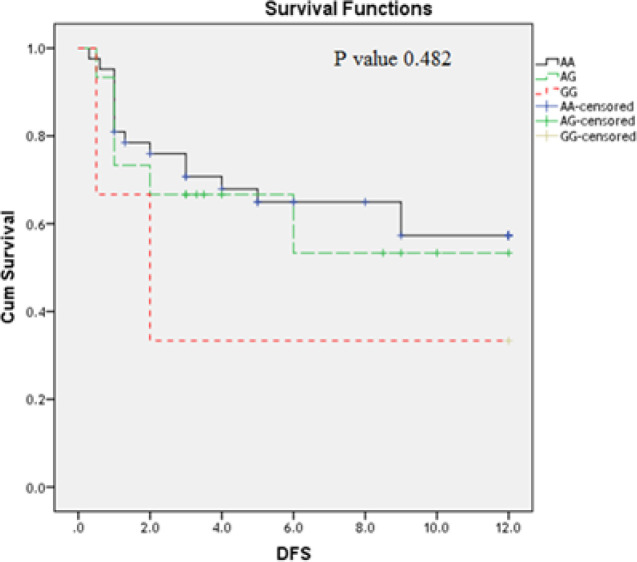
Impact of *TLR4 Asp299Gly* Genotype Variants on AML DFS (Months)

**Table 5:** Impact of *TLR2 Arg753Gln* and *TLR4 (Thr399Ile & Asp299Gly) *Genotype Variants Regarding Sepsis among AML Patients

Genotypes		AML		Relative risk in AML			
		Absence of sepsis	Presence of Sepsis	OR	95%CI		P value
		(N=100)	(N=20)				
TLR2 Arg753Gln	AA	90	5	1	-	-	R
	AG	6	9	21.37	3.28	139.1	0.001
	GG	4	6	30.33	1.31	697.6	0.032
	A	186	19	17.64	3.632	85.74	<0.001
	G	14	21				
TLR4 Thr399Ile	CC	75	10	1	-	-	R
	CT	20	13	4.57	1.02	20.34	0.046
	TT	5	2	2.28	0.124	41.98	0.577
	C	170	33	2.727	0.869	8.55	0.04
	T	30	17				
TLR4 Asp299Gly	AA	85	11	1	-	-	R
	AG	10	2	10.28	1.73	60.9	0.01
	GG	5	7	2.57	0.14	47.01	0.524
	A	180	25	4.318	1.209	15.42	0.024
	G	20	15				

CI, Confidence interval; OR, odds ratio

**Table 6:** Impact of *TLR2 Arg753Gln* and *TLR4 (Thr399Ile & Asp299Gly)* Genotype Variants Regarding Pneumonia among AML and ALL Patients

Genotypes		AML		Relative risk in AML			
		Absence of pneumonia	Presence of pneumonia	OR	95%CI		P value
		(N=100)	(N=20)				
TLR2 Arg753Gln	AA	57	15	1	-	-	R
	AG	39	2	0.187	0.02	1.739	0.148
	GG	4	3	3.75	0.293	47.99	0.309
	A	153	32	0.98	0.299	3.215	0.973
	G	47	8				
TLR4 Thr399Ile	CC	88	2	1	-	-	R
	CT	8	16	66	6.03	722	0.001
	TT	2	2	22	0.719	672.8	0.0765
	C	188	20	9.4	2.708	32.62	<0.001
	T	12	20				
TLR4 Asp299Gly	AA	84	5	1	-	-	R
	AG	11	13	17.11	2.79	104.7	0.002
	GG	5	2	7.33	0.356	150.7	0.196
	A	179	23	6.5	1.85	22.8	0.003
	G	21	17				

## Results

*Patients characteristics*

The patient’s characteristics are shown in Table 1. 

*Association between TLR polymorphism and AML*

No significant differences in genotype or alleles frequency regarding *TLR2 Arg753Gln*, *TLR4 Asp299Gly* and *TLR4 Thr399Ile* polymorphisms in AML and healthy controls. (P>0.05 for all) (Tables 2-4). All three SNPs were in accordance with Hardy–Weinberg equilibrium (*χ*^2^ test). 

Impact of *TLR2 Arg753Gln* and *TLR4 (Thr399Ile and Asp299Gly)* genotype variants on development of sepsis

*TLR2 Arg753Gln:* AG genotypes and A allele;* TLR4 Asp299Gly: CT* genotype and C allele; *TLR4 Thr399Ile AG* genotype and A allele showed significant higher risk to sepsis as compared to other genotypes and alleles (Table 5). Pneumonia was significantly associated with *TLR4 Asp299Gly AG* genotype and A allele and *TLR4 Thr399Ile CT* genotype and T allele; but there is no association was found with *TLR 2 TLR2 Arg753Gln *polymorphism (Table 6). 

*Survival Analysis*

At the end of follow up period (12 months), OS of studied AML patients estimates 49.7% at 6 months interval and 34.9% at 12 months interval (Table 7). The impact of *TLR* polymorphism on the AML patient’s overall survival was studied by Kaplan Mire curve. AML patients have *TLR2 (Arg753Gln) GG* genotype had the shortest OS; followed by those have AG and GG genotypes (P=0.001) (Figure 1); while *TLR4 (Thr399Ile and Asp299Gly)* polymorphism did not affect the OS (P=0.06; 0,104 respectively) (Figures 2 and 3). 

Moreover; the studies on the DFS revealed that AML patients have *TLR2* polymorphic AA genotype(P=0.002) (Figure 4) and those who have *TLR4 Thr399Ile CC* genotype have the longest DFS as compared to the other genotypes CT; TT (Figure 5) (P= 0.015); while TLR4 Asp299Gly didn’t have an association with DFS (P=0.48) (Figures 6).

## Discussion

Genetic variations of the pathogen recognition system are supposed to be responsible for individual differences concerning the response to infectious stimuli. In detail, a series of SNPs affecting key molecules of innate immunity including surface molecules such as *TLR2*, *TLR4, TLR5* and different variants of the mannose-binding lectin 2 or intracellular downstream molecules as the IL-1 receptor-associated kinase and the TIR-domain-containing adaptor protein have been attributed with an increased risk of infections (Ahmad-Nejad et al,. 2011; Kumpf et al., 2010; Sánchez-Canossa et al., 2016).

Herein; we investigated the impact of the *TLR2* and *TLR4* polymorphisms on the susceptibility of AML patients to infection as well as patient’s outcome. We found that AML patients who have *TLR2 Arg753Gln: AG* genotypes and A allele; *TLR4 Asp299Gly*: CT genotype and C allele; *TLR4 Thr399Ile AG *genotype and A allele showed significant higher association to sepsis and pneumonia as compared to other genotypes and alleles. This finding is consistent with that reported by Schnetzke et al., (2015) who reported that The presence of the *TLR2 Arg753Gln* polymorphism was significantly associated with pneumonia in AML patients and that the cosegregating *TLR4* polymorphisms *Asp299Gly *and *Thr399Ile* were independent risk factors for the development of both sepsis and pneumonia. Furthermore; *TLR2* polymorphism has been demonstrated as a risk factor for development of severe infections in critically ill patients from a surgical intensive care unit (Ahmad-Nejad et al., 2001) and higher rates of infection recurrence and initial septic shock in liver-transplant recipients developing gram-positive infections (Lee et al., 2001). In addition; the results are also supported by that found by Schnetzke et al., (2015); Avdonina et al., (2017) who stated the association* TLR4* polymorphisms were independent risk factors for the development of both sepsis and pneumonia in AML patients.

It has been reported that *TLR4* polymorphisms are present in 10% of Caucasian populations and are reported to have a positive correlation with susceptibility to several infectious diseases (including gram-negative sepsis), atherosclerosis, asthma, malaria and also Helicobacter pylori-induced gastric cancer (Mockernut et al., 2006; Hold et al., 2007). Two studies (Agnese et al., 2002; Lorenz et al., 2002) revealed that the risk of septic shock due to infection by Gram-negatives is increased in the presence of these SNPs. 

The impact of *TLR* polymorphism on the AML patient’s overall survival was studied by Kaplan Mire curve. AML patients who have *TLR2 (Arg753Gln) GG* genotype had the shortest OS; followed by those have AG and AA genotypes (P=0.001); while *TLR4 (Thr399Ile and Asp299Gly) *polymorphism did not affect the OS (P=0.06; 0,104 respectively). These findings are inagreement with that reported a previous study (Schnetzke et al., 2015). 

Moreover; the studies on the DFS revealed that AML patients have *TLR2* polymorphic AA genotype(P=0.002) and those who have* TLR4 Thr399Ile CC *genotype have the longest disease free survival as compared to the other genotypes CT; TT ; while *TLR4 Asp299Gly* didn’t have an impact on OS . No previous study was evaluated the impact of these polymorphism on the AML patient’s outcome. 

In conclusion, *TLR2 Arg753Gln* (AG; GG genotype) polymorphisms are associated shortest OS and DFS. Moreover; significant association between TLR2 polymorphisms, *TLR4 Arg753Gln* polymorphisms and risk of infections in AML patients was documented.
